# Spotting, Transcription and *In Situ* Synthesis: Three Routes for the Fabrication of RNA Microarrays

**DOI:** 10.1016/j.csbj.2019.06.004

**Published:** 2019-06-27

**Authors:** Jory Lietard, Mark M. Somoza

**Affiliations:** Institute of Inorganic Chemistry, Faculty of Chemistry, University of Vienna, Althanstraße 14, UZA II, 1090 Vienna, Austria

**Keywords:** Microarray, Photolithography, Spotting, Transcription, *in situ* RNA synthesis, RNA, Phosphoramidites, Solid-phase synthesis

## Abstract

DNA microarrays have become commonplace in the last two decades, but the synthesis of other nucleic acids biochips, most importantly RNA, has only recently been developed to a similar extent. RNA microarrays can be seen as organized surfaces displaying a potentially very large number of unique sequences and are of invaluable help in understanding the complexity of RNA structure and function as they allow the probing and treatment of each of the many different sequences simultaneously. Three approaches have emerged for the fabrication of RNA microarrays. The earliest examples used a direct, manual or mechanical, deposition of pre-synthesized, purified RNA oligonucleotides onto the surface in a process called spotting. In a second approach, pre-spotted or *in situ*-synthesized DNA microarrays are employed as templates for the transcription of RNA, subsequently or immediately captured on the surface. Finally, a third approach attempts to mirror the phosphoramidite-based protocols for *in situ* synthesis of high-density DNA arrays in order to produce *in situ* synthesized RNA microarrays. In this mini-review, we describe the chemistry and the engineering behind the fabrications methods, underlining the advantages and shortcomings of each, and illustrate how versatile these platforms can be by presenting some of their applications.

## 1 Introduction

The function and biological role of RNA is a story far more complex than that of DNA and is still being fully explored [1,2]. Not only is it a carrier of genetic information, in viruses and perhaps in the earliest forms of life as well (the RNA world hypothesis), it is also a molecule of formidable conformational plasticity, a result of the hydrogen bond network woven by 2′-OH groups, yielding a wide spectrum of tridimensional structures that can impart function to the RNA. The functional landscape of RNA is just as diverse, from ribosomal RNA to ribozymes [3], riboswitches [4], protein-binding [5] and small molecule binding RNA aptamers [6]. Small RNAs, effectors in the interference (RNAi) or CRISPR/Cas systems and fundamental defense mechanisms in eukaryotic and prokaryotic life [7,8], have been quite recently discovered and further underline the rich and complex biological tasks of RNA [9,10]. However, understanding function is not a straightforward process, as it usually requires the study of multiple sequence variants or a systematic mutational analysis. And improving function, for instance with more efficient siRNAs, can only be accessed through careful chemical modifications [11]. In the context of our quickly expanding understanding of the chemical and biological nature of RNA, high-throughput approaches for the study of RNA structure and function are highly desirable. RNA-seq belongs to such a category, as it allows for transcriptome profiling on a massive scale, can inform on alternative splice sites and on single nucleotide polymorphisms, and has also delivered on the identification of changes in transcriptional levels between cell populations [12,13], a hallmark application of DNA microarrays.

DNA microarrays refer to libraries of DNA sequences chemically attached to a single support, often a standard microscope slide, with each sequence being precisely positioned on the surface [14]. This sequence library can then be hybridized with fluorescently-labeled cDNA originating from the reverse-transcription of mRNAs. The resulting surface fluorescence intensities are proportional to the abundance of a particular transcript. Comparing the fluorescence signals from two different samples can reveal differences in gene expression levels [15]. Beyond Watson-Crick-based DNA/DNA pairing, DNA microarrays have become sensing platforms as well as tools to investigate DNA-binding proteins and are rapidly gaining interest as a cost-effective method for DNA synthesis *en route* towards DNA-based digital information storage solutions [16,17] [18]. In practice, DNA microarrays are either spotted or fabricated *in situ*. In spotting, droplets of individual DNA sequences are manually or mechanically deposited onto the slide and functional groups at the surface and on the 5′ or 3′ extremity of the oligonucleotide react together to create a covalent attachment. On the other hand, *in situ* fabrication uses the slide as a support for oligonucleotide synthesis, which typically proceeds *via* cycle-based phosphoramidite chemistry. Regardless of the fabrication process, DNA arrays offer the ability to assay, in parallel, the chemistry, the kinetics or the binding affinities of thousands of variants to a given target in a single experiment, a feat which highlights the great potential of RNA microarrays as a high-throughput technique. However, adapting the fabrication methods of DNA microarrays to RNA has not been a straightforward process and remains technically and chemically challenging, partly because of the instability of the RNA molecule, requiring careful handling, and partly because of the non-trivial protection/deprotection strategies associated with RNA phosphoramidite chemistry. Still, the last 15 years have seen the birth and development of the three main approaches for the preparation of RNA microarrays: spotting, DNA transcription and *in situ* photolithography. In this mini-review, we intend to present and summarize the design and chemical processes behind these three routes as well as provide an overview of the realm of applications.

## 2 Fabrication Methods

### 2.1 Spotting

**Fig. 1:**
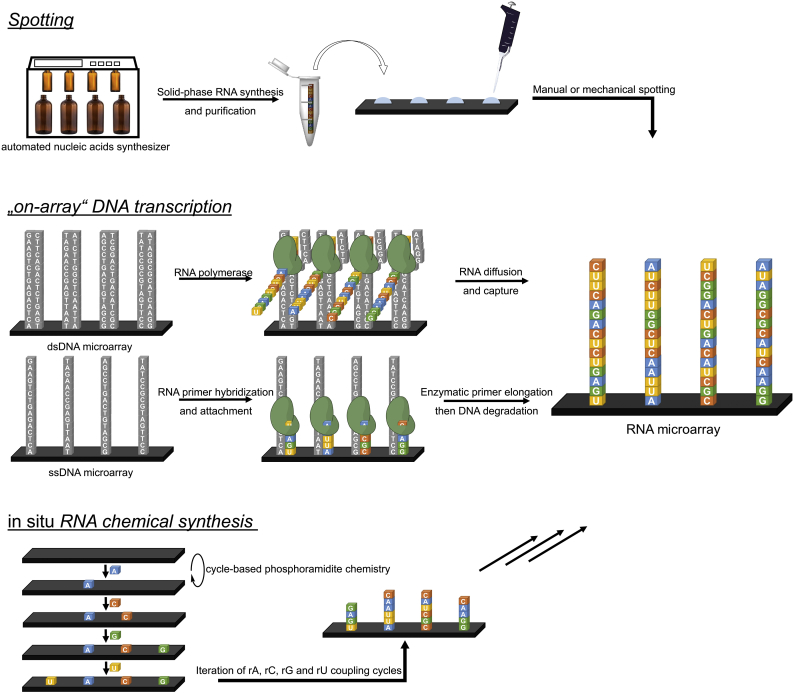
Schematic representation of the three different approaches to the fabrication of RNA microarrays: spotting, transcription of DNA microarrays, and *in situ* RNA synthesis using phosphoramidite chemistry. DNA transcription can be performed from ssDNA or dsDNA. In ssDNA, a small RNA primer must first hybridize and be covalently bound to the surface or the DNA template, and primer elongation leads to the synthesis of ssRNA. In dsDNA, the transcribed RNA is initially in the solution phase, but can diffuse and be captured in a different region of the array surface, or in a separate surface altogether. DNA nucleotides are shown in grey, and RNA nucleotides in colour. DNA and RNA sequences are arbitrary and serve to illustrate the fabrication processes.

**Fig. 2:**
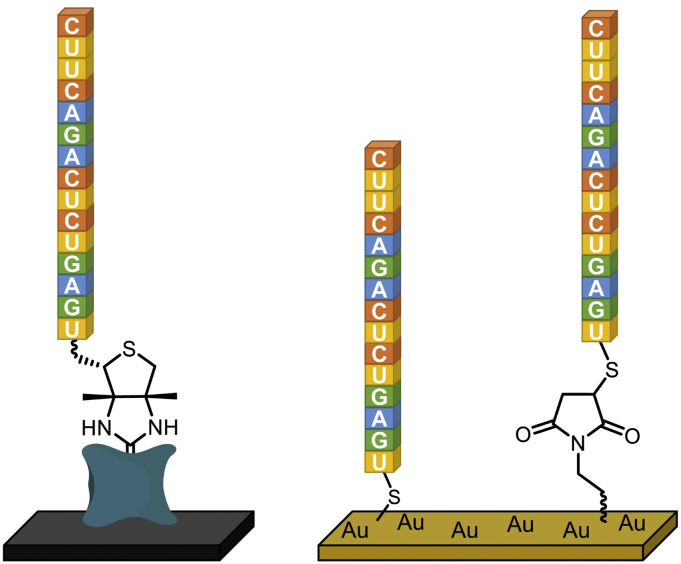
Two of the main spotting approaches for RNA microarrays (the given sequence is purely illustrative): left, immobilization of the RNA molecule through formation of a strong biotin-streptavidin bond, right, thiol or thiotriphosphate-containing RNA can be spotted either directly onto the gold layer or indirectly attached to gold by reacting with a maleimide functional group. (For interpretation of the references to colour in this figure legend, the reader is referred to the web version of this article.)

Spotting is perhaps the simplest and most straightforward approach for the fabrication of RNA microarrays. It is indeed through spotting that the first DNA microarrays were produced, and it therefore comes as no surprise that the earliest attempts at arraying RNA onto a surface were also conducted using pre-synthesized RNA oligonucleotides. In so doing, the RNA is synthesized *ex situ* on solid support using standard phosphoramidite and 2′-*O*-TBDMS chemistries then purified, or purchased from a commercial source directly in pure form. Pre-synthesis also allows for the introduction of chemical modifications at the base, sugar or internucleotidic levels. Alternatively, RNA may be obtained by transcription of the template DNA sequence. In essence, spotting grants control over RNA sequence and purity, a probable necessity in the conception of the first RNA microchips, and confines all chemical steps to a single reaction: attaching the RNA molecule to the surface (Fig. 1). The extremely high affinity of biotin for streptavidin can be harnessed and used as a fixing point for oligonucleotides onto commercially-available surfaces pre-coated with streptavidin. Biotin is conveniently incorporated at the 5′ or 3′ end of RNA oligonucleotides during synthesis using the corresponding phosphoramidite, or added to a nucleotide monophosphate in transcription. In a buffered aqueous solution, 5′-biotinylated RNA oligomers were spotted onto streptavidin-coated slides using manual or automated arrayers [[19], [20], [21]], delivering volumes as little as 3 nL and resulting in spot sizes of 500–700 μm in this case. Spotting parameters such as buffer, humidity and temperature are deciding factors of the overall spot shape and uniformity [22]. Another approach for spotting is to exploit the high affinity of sulfur for gold with thio-modified oligonucleotides and gold surfaces (Fig. 2). In one of the earliest reports on the preparation of RNA microarrays, dsDNA was first transcribed into RNA starting with a 5′-*O*-(3-thiotriphosphate) guanosine ribonucleotide, and the resulting thio-containing RNA was immobilized onto a gold surface [23]. Multiple RNA sequences were also arrayed onto polystyrene microtiter plates pre-coated with gold. In a slightly different strategy, gold surfaces were used in combination with thiol-modified RNA oligonucleotides, but the attachment chemistry relied on the reaction of 5′-thiol functions with electrophilic maleimide groups, which were added as a reactive layer on an amine-terminated gold surface using a bifunctional maleimide-succinimide cross-linker [24]. Spotting itself was accomplished using a picopump delivering ≈40 nL of a buffered, thiol-containing RNA solution. An interesting variation on this theme starts with the spotting on gold-coated slides of 3′-SH DNA molecules terminated with a 5′-phosphate group. Through templated ligation, a 3′-OH RNA oligonucleotide is ligated atop the 5′-p spotted DNA sequence, thus forming RNA-DNA spotted microarrays [[25], [26], [27]]. Finally, an ingenious process for spotting RNA microarrays starts from pre-printed DNA on surfaces. In one instance, spotted DNA arrays are first hybridized to their complementary RNA strand carrying a biotin group at their 3′-end. A streptavidin-coated surface is then brought into conformal contact with the DNA array, at which point the biotinylated RNA is captured and transfers over to the streptavidin slide [28]. An alternative version of this process begins with the photolithographic etching of silicon wafers in order to produce micro-stamps, which are then coated with a complex of positively-charged dendrimers and RNA, bound together through electrostatic interactions. An aldehyde-coated gold surface then comes into contact with the master slide only at the un-etched regions (micro-contact printing), capturing the RNA oligonucleotide *via* the formation of a Schiff base [29]. These “sandwich” methods for RNA transfer and immobilization carry the additional advantage of keeping the same density and spot size as the master surface which, in the spotted DNA master, displayed 2500 individual spots 70 μm in size and, in the etched surface example, may yield RNA patterns only nanometers wide.

### 2.2 “On-array” DNA Transcription into RNA

An entirely different approach to RNA array fabrication takes advantage of the rapidity and high-fidelity of enzymes to polymerize RNA from a DNA template, an approach that minimizes RNA handling and instead relies on a starting deoxyribose array, which is easier to manipulate, cheaper to synthesize, and already available in high-density. Indeed, the history of DNA microarray fabrication is at least a decade older than that of RNA and similarly started with spotting pre-synthesized DNA oligonucleotides, but the technology gained momentum as the phosphoramidite chemistry was successful ported to the glass surface, allowing for hundreds, thousands, then hundreds of thousands of different DNA sequences to be synthesized in parallel, and *in situ* [[30], [31], [32]]. DNA arrays become surfaces where “on-array” transcription can be carried out, resulting in the same number of unique RNA transcripts and at the same density, provided the transcribed RNA can be immobilized on the surface as well. In fact, the capture of newly-transcribed RNA oligonucleotide is a central aspect of the method. One possibility is to let the RNA diffuse in the solution phase and be captured elsewhere on the surface. In this approach, 5′-amino modified ssDNA was first spotted on a gold surface functionalized with glutamic acid groups through amide bond formation using 1-ethyl-3-(3-dimethylaminopropyl)carbodiimide (EDC) as a coupling agent. The surface is equipped with a microfluidic channel. After addition of the complementary DNA strand, the dsDNA is transcribed with T7 RNA polymerase and the nascent RNA transcript diffuses along the microfluidic channel, to be captured downstream by a short DNA strand *via* hybridization [33]. Alternatively, the RNA may be captured on a separate slide. In a recent report, spotted 5′-biotinylated dsDNA sequences underwent “on-array” RNA transcription using T7 RNA polymerase, the DNA template encoding in addition for an aptamer construct recognizing and binding to streptavidin or tobramycin. Thus, when a second, separate surface coated with streptavidin or with the antibiotic tobramycin was brought over to the dsDNA slide into direct contact, the aptamer-messenger RNA diffused over to the second slide and was captured by the respective aptamer ligand [34]. Up to eight different RNA sequences were transcribed at once and then captured at precise and well-defined locations, with RNA spot sizes varying between 150 and 300 μm.

Diffusion and capture being an additional step in the process of RNA array fabrication and governed by its own set of physical and chemical rules, forcing the RNA to remain where it was originally transcribed on the surface appears convenient. However, in this approach, where template and transcript do not completely dissociate, the fate of the template DNA needs to be addressed. One solution is to leave the dsDNA template intact, all the while keeping the transcribed RNA loosely bound to its template. This was recently shown to be a viable method for RNA array fabrication on a sequencing flow cell as a platform for “on-array” transcription [35,36]. A library of dsDNA templates was first sequenced on a standard Illumina flow cell, which provided positional information for each of the DNA sequences on the surface of the cell. The complement of the DNA strand attached to the surface was terminated at the 5′-end with biotin and the addition of streptavidin created a large proteinic complex akin to a roadblock. When the RNA polymerase reaches this roadblock, the enzyme stalls and the nascent RNA transcript remains tethered to the dsDNA-polymerase-streptavidin complex. In so doing, the authors reached an impressive density of 10^7^ RNA sequences on the flow cell. An adaptation of this procedure uses the Tus protein, a known replication terminator, to halt RNA transcription once the RNA polymerase reaches this block. Due to incomplete polymerization, the RNA remains bound to the transcription complex [37]. Flow cell density allowed for up to 12,000 different RNA sequences to be produced in a single array format.

Template degradation is perhaps the closest step to an RNA-only microchip, but in order for this step to successfully proceed, the transcribed RNA must be bound to the surface independently from its DNA template, all the while avoiding diffusion into the solution phase so as to retain the uniformity and the spatial organization of the template DNA array. This challenge can be solved using proximity and primer elongation. Single-stranded DNA microarrays are first synthesized *in situ* using a photolithographic approach, which uses UV light to trigger the removal of the photosensitive NPPOC group at the 5′-end of the growing oligonucleotide strand (*vide infra*) [31]. After synthesis of the DNA template with phosphoramidite chemistry, exposure of the same area to an acidic solution deblocks acid-sensitive sites, where an RNA primer is synthesized using standard, DMTr-protected reverse RNA amidites. After DNA and RNA deprotection, the RNA primer can hybridize to its complementary sequence on the DNA template since they were both synthesized on the same 14 μm-wide square area (also called “feature”). Using T7 RNA polymerase, the primer is first elongated and the DNA template is ultimately degraded with Turbo DNase, thus affording a DNA-free RNA microarray whose sequence density is as high as that of template DNA [38,39]. With *in situ* synthesis being a prime example of a chip fabrication process compatible with high density and high complexity, “on-array” transcription of *in situ*-synthesized DNA array can be seen as a real route towards high-density RNA microarrays.

### 2.3 *In Situ* RNA Chemical Synthesis

**Fig. 3:**
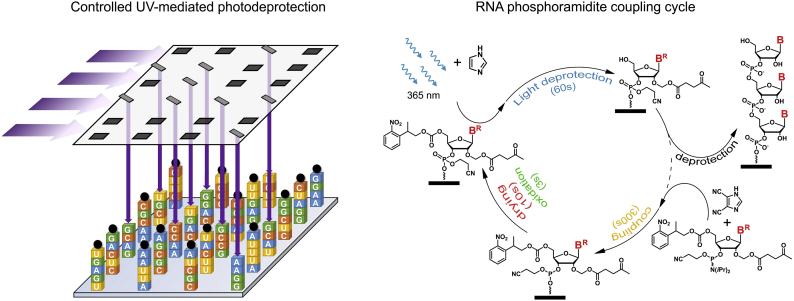
Left: an illustration of the photolithography process for RNA array fabrication. Incoming UV light (purple arrows) reflects onto the surface only when the corresponding micromirrors are properly tilted in the “ON” position. The reflected UV light triggers the deprotection of the NPPOC group (black ball) at the 5′ end of the growing oligonucleotide strand. Right: chemical coupling cycle of an RNA phosphoramidite. After UV light deprotection as shown in the left part of the figure, the amidite couples to the 5′-OH oligonucleotides using dicyanoimidazole as a coupling agent, followed by drying of the surface, and oxidation of the phosphite triesters into phosphotriesters. Microarray synthesis then enters a new cycle with the photodeprotection of selected NPPOC groups. After synthesis, RNA oligonucleotides are deprotected in two steps under mildly basic conditions. (For interpretation of the references to colour in this figure legend, the reader is referred to the web version of this article.)

The protocols for solid-phase synthesis of DNA oligonucleotides have long been adapted to the parallel synthesis of thousands of different DNA molecules on flat substrates, with photolithography, electrochemistry and inkjet printing three means of addressing precise and well-defined locations on a substrate. And technological improvements in the miniaturization of mirrors, electrodes and printing heads further increases the sequence complexity that a single flat surface can hold. Those improvements are made over the rock-solid foundation of DNA synthesis *via* phosphoramidite chemistry, an extremely robust, fast and efficient coupling reaction that can proceed just as efficiently on RNA phosphoramidites, albeit with longer coupling times. However, the synthetic protocols for RNA oligonucleotide elongation on solid-phase are not directly translatable to microarrays and the issue simply boils down to the choice of protecting group for the 2′ hydroxyl. In solid-phase synthesis, a bulky silyl group protects the 2′-OH and is post-synthetically removed with fluoride-containing organic salts or acids, an incompatible approach for microarray fabrication on glass as it would corrode the surface and wash away the synthesized RNA. The silyl protection strategy could be suitable for *in situ* RNA synthesis on non‑silicon based substrates, such as glassy carbon, PTFE or other polymers, but has not been pursued yet. Alternative 2′-protecting groups exist, but they are neither as universal as the standard TBDMS moiety nor are the corresponding phosphoramidites necessarily compatible with array synthesis. One of those alternatives relies on the use of the levulinyl (lev) group as a hydroxyl protection strategy, transforming the OH into a ketoester that can easily be removed under mild conditions with hydrazine hydrate: hydrazine first reacts with the ketone function to form a hydrazone, and the second amine can then react with the nearby ester function, releasing the hydroxyl group and a hydropyridazine as a byproduct [40]. A set of RNA phosphoramidites appropriately protected with levulinyl groups was recently prepared for solid-phase synthesis [41], and the final tweak was the introduction of an acetal spacer between the hydroxyl and the levulinyl that prevents the migration of the ester function from the 2′ to the 3′ position, an issue particularly relevant during phosphoramidite synthesis. Ribonucleosides, grafted with an acetal-levulinyl ester (ALE) at the 2′-OH and protected at the exocyclic amines of the nucleobases with lev or dimethylformamidine were made UV-photosensitive by attaching a nitrophenylpropyloxycarbonyl (NPPOC) at the 5′-OH [42]. RNA oligonucleotides were then synthesized *in situ* on a silanized glass slide by maskless photolithography, where an array of small micromirrors, electronically controlled by a computer, can be tilted into a position so as to reflect the incoming UV light onto the surface (Fig. 3). Oligonucleotides on the UV-exposed features are photodeprotected, *i.e.* the NPPOC group is removed, allowing for coupling with the next phosphoramidite. The micromirror device can hold from >768,000 up to 2 million individually addressable mirrors on a small area (1.4–2.5 cm^2^), and with the layout of tilted mirrors being imaged 1:1 onto the substrate, the microscope slide can therefore contain as many features as there are mirrors and within the same area. Therefore, photolithography can be regarded as a high-density array fabrication method. We first reported on the synthesis of rU and rA homopolymers [[42], [43], [44]], but we recently extended our synthesis capabilities to mixed-base RNA, 30-nt in length, and to >250,000 different RNA sequences on the same surface [45].

## 3 Applications

In many instances, a simple hybridization to the RNA with a fluorescently-labeled complementary strand is necessary and sufficient to indicate correct spotting/transcription/synthesis. But the usefulness of RNA microarrays goes beyond that of traditional DNA microarrays, extending for instance, to the study of biologically-relevant interactions such as ribozymes and enzymes, aptamer and RNA-binding proteins.

### 3.1 Kinetics and Enzymatic Degradation

RNases are a class of enzymes that have been heavily investigated on RNA microarrays. RNA oligonucleotides ligated onto spotted DNA gold slides were exposed to RNase S and were shown to be correctly degraded using Surface Plasmon Resonance Imaging (SPRI) [25]. When the RNA was first hybridized to its complementary DNA strand, treatment of the slide with RNase H led to degradation of the RNA, regenerating the 5′-phosphorylated DNA slide, able to undergo further rounds of RNA ligation. SPRI also helped to monitor the degradation of spotted RNA with RNase H, and the kinetics of RNA degradation were shown to be dependent on the concentration of the complementary DNA strand in solution and, ultimately, to the surface coverage of RNA molecules by DNA (or fractional surface coverage by hybridization) [24]. In *in situ* synthesized microarrays, an RNase A assay on sequences containing a single rU monomer flanked by a varying number of dA nucleotides revealed kinetics and sequence preferences comparable to solution-phase data, *i.e.* substrates with a greater number of pyrimidine nucleobases around the rU insert were better recognized and cleaved by the enzyme [43]. With RNase HII, an enzyme responsible for removing misincorporated ribonucleotides during DNA copying, an assay was performed on a library of DNA hairpins containing a single RNA nucleotide. Base permutations around and at the site of RNA insertion yielded a library of 1024 unique sequences (5^4^ permutations of a 5-nt long region). RNase HII-mediated enzymatic degradation performed significantly better for substrates containing the 5′-dC-rC-3′ motif, but appeared to be independent of the nature of the DNA base 3′ to the RNA incorporation, which is the site of 2′-deoxy sensing by the enzyme. RNase H also recognizes and cleaves RNA oligos synthesized *in situ*, while leaving the corresponding DNA sequences intact [45].

Enzymatic properties have been observed and described in very specific DNA and RNA sequences. The well-known RNA-cleaving 10–23 DNAzyme was used to degrade RNA oligonucleotides transcribed from high-density DNA arrays, while DNA sequences remained untouched. Similarly, RNase A degraded transcribed RNA but not the template DNA [38]. Ribozymes have also been studied on surfaces. A series of seven different radiolabeled RNA “switches” were spotted on a gold substrate and upon binding of a ligand to an allosteric receptor (“switch”), the second part of the RNA sequence folds into the tridimensional conformation of the self-cleaving hammerhead ribozyme. Switch triggers were chosen among various families: from ions and heterocyclic bases to second messengers. After binding of the receptor to the correct analyte, self-cleavage of the ribozyme region was demonstrated with the loss of ^32^P radioactivity. Taken together, these assays not only serve to confirm the integrity and identity of the RNA, regardless of the microarray fabrication approach, they also verify the functionality and the accessibility of immobilized RNAs to small molecules, nucleic acids and enzymes.

### 3.2 RNA Aptamers and RNA-binding Proteins

A fair number of arrayed RNA oligonucleotides have been used in non-destructive binding experiments, most notably to proteins, in order to highlight the ability of the microchip to act as a detection platform. RNA arrays were successfully used to detect thrombin [27,33], vascular endothelial growth factor as well as basic fibroblast growth factor (VEGF and bFGF, respectively) [19,27,33], lysozyme [20,21], and ricin [21] in solution by spotting the corresponding RNA aptamer sequence or transcribing it from the template DNA. Using fluorescence as the signal recording method, the dynamic range of detection was shown to span several orders of magnitude, reaching low nanomolar or even femtomolar concentration of the target protein in solution. Importantly, immobilized RNA aptamers were found to bind to their respective targets when diluted into biological samples or cell lysates [19,20]. An exhaustive study of the influence of binding assay and spotting parameters on lysozyme and ricin binding was also undertaken, and the authors remarked that surface coating and assay buffers were the most likely to affect signal intensity and signal-to-noise ratio. SPRI measurements on spotted or transcribed RNA on gold surfaces against thrombin, VEGF, or the protein factor IXa allowed not only measurement of surface adsorption constants, but also distinction between the binding of two different RNA aptamers to their respective targets as well as between sequence variants of the same aptamer [26,27,33]. Small molecule-binding RNA aptamers have been prepared *via* transcription of spotted DNA arrays and assayed in presence of the triarylmethane dye malachite green [34]. The fluorescence of malachite green increases >2000 fold upon binding to the RNA aptamer and the spots containing the correct, transcribed and immobilized RNA sequence indeed lit up in presence of the dye.

RNA microarrays transcribed from immobilized DNA on Illumina flow cells generated a colossal amount of data, owing to the high density and complexity of the available sequence space, from which RNA-protein dissociation constants could be extracted. Binding of a wild-type RNA aptamer to either the coat protein of the MS2 bacteriophage, the GFP protein or the negative elongation factor E (NELFe), or the Vts1 protein afforded *K*_d_ values comparable to earlier literature measurements [[35], [36], [37]], an important comparison in order to give credence to binding assays performed at the interface between surface and solution. The large number of RNA sequence variants on those platforms was also used to perform a deep mutational analysis, revealing the effect of single point mutations on the binding affinity and deciphering novel binding motifs. These large-scale assays represent a significant step towards a better understanding of the mechanisms of RNA-protein interactions.

## 4 Summary and Outlook

Herein lies a real advantage of microarrays over other high-throughput methods; they allow for the parallel study (*i.e.* a single experiment) of sequence variations in presence of a given target, with each nucleic acids construct fixed and immobilized in a known and well-defined location that no other construct occupies, and the assay provides a complete overview of the reaction landscape, from poor substrates and poor binders to excellent substrates and strong aptamers. RNA microarrays certainly find their *raison d'être* in being interrogation platforms for a multitude of RNA·ligand interactions, but in order to perform and deliver a comprehensive understanding of binding/reaction mechanisms, RNA arrays need to be high-density. Here, the three approaches described in this review can be reduced down to two categories: one where the surface is a receptacle for pre-synthesized RNA oligonucleotides, and one where the surface actively participates in RNA synthesis either directly or through DNA. The first category refers to spotting and is an excellent approach to verify the functionality and usefulness of RNA molecules covalently attached to a slide, but is low-density due to the need for a pre-synthesized oligonucleotide for each spot on the surface. Conversely, *in situ* RNA synthesis, chemical or enzymatic, is a high-density fabrication method as it yields hundreds of thousands and up to several millions of variants and, *de facto*, appears to be the only way to probe a large enough permutation space. Synthetic and technical improvements may yet increase this capacity by a few orders of magnitude. However, high-density RNA arrays may still be insufficiently complex when it comes to finding and selecting entirely novel RNA structures that bind a chosen target, as SELEX experiments typically start with combinatorial libraries containing >10^15^ sequences. Microarrays also currently suffer from a dearth of modified nucleosides available for array fabrication —and microarrays are ideal tools to perform systematic substitutions between natural and synthetic analogs— [46] but the situation is expected to improve as additional photoprotected RNA phosphoramidites and synthetic polymerases become more accessible.

## Acknowledgements

The Austrian Science Fund (FWF P23797, P27275 and P30596) and the Swiss National Science Foundation (PBBEP2_146174) are gratefully acknowledged for the support and the funding of the work on RNA photolithography presented in this review.

## References

[1] Sharp P.A. (2009). The centrality of RNA. Cell.

[2] Breaker R.R., Joyce G.F. (2014). The expanding view of RNA and DNA function. Chem Biol.

[3] Wilson T.J., Liu Y.J., Lilley D.M.J. (2016). Ribozymes and the mechanisms that underlie RNA catalysis. Front Chem Sci Eng.

[4] Serganov A., Nudler E. (2013). A decade of riboswitches. Cell.

[5] Hentze M.W., Castello A., Schwarzl T., Preiss T. (2018). A brave new world of RNA-binding proteins. Nat Rev Mol Cell Biol.

[6] Dolgosheina E.V., Unrau P.J. (2016). Fluorophore-binding RNA aptamers and their applications. WIREs RNA.

[7] Wilson R.C., Doudna J.A. (2013). Molecular mechanisms of RNA interference. Annu Rev Biophys.

[8] Barrangou R., Marraffini L.A. (2014). CRISPR-Cas systems: prokaryotes upgrade to adaptive immunity. Mol Cell.

[9] Cech T.R., Steitz J.A. (2014). The noncoding RNA revolution-trashing old rules to forge new ones. Cell.

[10] Strobel E.J., Watters K.E., Loughrey D., Lucks J.B. (2016). RNA systems biology: uniting functional discoveries and structural tools to understand global roles of RNAs. Curr Opin Biotechnol.

[11] Deleavey G.F., Damha M.J. (2012). Designing chemically modified oligonucleotides for targeted gene silencing. Chem Biol.

[12] Kukurba K.R., Montgomery S.B. (2015). RNA sequencing and analysis. Cold Spring Harb Protoc.

[13] Wang Z., Gerstein M., Snyder M. (2009). RNA-Seq: a revolutionary tool for transcriptomics. Nat Rev Genet.

[14] Bumgarner R. (2013). Curr Protoc Mol Biol.

[15] Karakach T.K., Flight R.M., Douglas S.E., Wentzell P.D. (2010). An introduction to DNA microarrays for gene expression analysis. Chemometr Intell Lab.

[16] Liu M.Y., Lou X.H., Du J., Guan M., Wang J., Ding X.F. (2012). DNAzyme-based fluorescent microarray for highly selective and sensitive detection of lead(II). Analyst.

[17] Tietjen J.R., Donato L.J., Bhimisaria D., Ansari A.Z. (2011). Sequence-specificity and energy landscapes of DNA-binding molecules. Methods Enzymol.

[18] Erlich Y., Zielinski D. (2017). DNA fountain enables a robust and efficient storage architecture. Science.

[19] McCauley T.G., Hamaguchi N., Stanton M. (2003). Aptamer-based biosensor arrays for detection and quantification of biological macromolecules. Anal Biochem.

[20] Collett J.R., Cho E.J., Lee J.F., Levy M., Hood A.J., Wan C. (2005). Functional RNA microarrays for high-throughput screening of antiprotein aptamers. Anal Biochem.

[21] Cho E.J., Collett J.R., Szafranska A.E., Ellington A.D. (2006). Optimization of aptamer microarray technology for multiple protein targets. Anal Chim Acta.

[22] Collett J.R., Cho E.J., Ellington A.D. (2005). Production and processing of aptamer microarrays. Methods.

[23] Seetharaman S., Zivarts M., Sudarsan N., Breaker R.R. (2001). Immobilized RNA switches for the analysis of complex chemical and biological mixtures. Nat Biotechnol.

[24] Goodrich T.T., Lee H.J., Corn R.M. (2004). Enzymatically amplified surface plasmon resonance imaging method using RNase H and RNA microarrays for the ultrasensitive detection of nucleic acids. Anal Chem.

[25] Lee H.J., Wark A.W., Li Y., Corn R.M. (2005). Fabricating RNA microarrays with RNA-DNA surface ligation chemistry. Anal Chem.

[26] Li Y., Lee H.J., Corn R.M. (2006). Fabrication and characterization of RNA aptamer microarrays for the study of protein-aptamer interactions with SPR imaging. Nucleic Acids Res.

[27] Li Y., Lee H.J., Corn R.M. (2007). Detection of protein biomarkers using RNA aptamer microarrays and enzymatically amplified surface plasmon resonance imaging. Anal Chem.

[28] Kim J.H., Crooks R.M. (2007). Parallel fabrication of RNA microarrays by mechanical transfer from a DNA master. Anal Chem.

[29] Rozkiewicz D.I., Brugman W., Kerkhoven R.M., Ravoo B.J., Reinhoudt D.N. (2007). Dendrimer-mediated transfer printing of DNA and RNA microarrays. J Am Chem Soc.

[30] Blanchard A.P., Kaiser R.J., Hood L.E. (1996). High-density oligonucleotide arrays. Biosens Bioelectron.

[31] Singh-Gasson S., Green R.D., Yue Y., Nelson C., Blattner F., Sussman M.R. (1999). Maskless fabrication of light-directed oligonucleotide microarrays using a digital micromirror array. Nat Biotechnol.

[32] LeProust E.M., Peck B.J., Spirin K., McCuen H.B., Moore B., Namsaraev E. (2010). Synthesis of high-quality libraries of long (150mer) oligonucleotides by a novel depurination controlled process. Nucleic Acids Res.

[33] Chen Y., Nakamoto K., Niwa O., Corn R.M. (2012). On-Chip synthesis of RNA aptamer microarrays for multiplexed protein biosensing with SPR imaging measurements. Langmuir.

[34] Phillips J.O., Butt L.E., Henderson C.A., Devonshire M., Healy J., Conway S.J. (2018). High-density functional-RNA arrays as a versatile platform for studying RNA-based interactions. Nucleic Acids Res.

[35] Buenrostro J.D., Araya C.L., Chircus L.M., Layton C.J., Chang H.Y., Snyder M.P. (2014). Quantitative analysis of RNA-protein interactions on a massively parallel array reveals biophysical and evolutionary landscapes. Nat Biotechnol.

[36] She R., Chakravarty A.K., Layton C.J., Chircus L.M., Andreasson J.O.L., Damaraju N. (2017). Comprehensive and quantitative mapping of RNA-protein interactions across a transcribed eukaryotic genome. Proc Natl Acad Sci U S A.

[37] Tome J.M., Ozer A., Pagano J.M., Gheba D., Schroth G.P., Lis J.T. (2014). Comprehensive analysis of RNA-protein interactions by high-throughput sequencing-RNA affinity profiling. Nat Methods.

[38] Wu C.-H., Holden M.T., Smith L.M. (2014). Enzymatic fabrication of high-density RNA arrays. Angew Chem Int Ed.

[39] Holden M.T., Carter M.C.D., Wu C.H., Wolfer J., Codner E., Sussman M.R. (2015). Photolithographic synthesis of high-density DNA and RNA arrays on flexible, transparent, and easily subdivided plastic substrates. Anal Chem.

[40] Ho T.L., Wong C.M. (1975). Hydroxyl protection by Levulinylation. Synth Comm.

[41] Lackey J.G., Sabatino D., Damha M.J. (2007). Solid-phase synthesis and on-column deprotection of RNA from 2 '- (and 3 '-) O-levulinated (lv) ribonucleoside monomers. Org Lett.

[42] Lackey J.G., Mitra D., Somoza M.M., Cerrina F., Damha M.J. (2009). Acetal Levulinyl Ester (ALE) groups for 2′-hydroxyl protection of Ribonucleosides in the synthesis of Oligoribonucleotides on glass and microarrays. J Am Chem Soc.

[43] Lackey J.G., Somoza M.M., Mitra D., Cerrina F., Damha M.J. (2009). In-situ chemical synthesis of rU-DNA chimeras on chips and enzymatic recognition. Chim Oggi-Chem Today.

[44] Lietard J., Kretschy N., Sack M., Wahba A.S., Somoza M.M., Damha M.J. (2014). Base-cleavable microarrays for the characterization of DNA and RNA oligonucleotides synthesized in situ by photolithography. Chem Commun.

[45] Lietard J., Ameur D., Damha M., Somoza M.M. (2018). High-density RNA microarrays synthesized in situ by photolithography. Angew Chem Int Ed.

[46] Lietard J., Abou Assi H., Gomez-Pinto I., Gonzalez C., Somoza M.M., Damha M.J. (2017). Mapping the affinity landscape of thrombin-binding aptamers on 2'F-ANA/DNA chimeric G-Quadruplex microarrays. Nucleic Acids Res.

